# The impact of hemodynamic variability and signal mixing on the identifiability of effective connectivity structures in BOLD fMRI

**DOI:** 10.1002/brb3.777

**Published:** 2017-07-20

**Authors:** Natalia Z. Bielczyk, Alberto Llera, Jan K. Buitelaar, Jeffrey C. Glennon, Christian F. Beckmann

**Affiliations:** ^1^ Donders Institute for Brain, Cognition and Behavior Nijmegen The Netherlands; ^2^ Radboud University Nijmegen Medical Centre Nijmegen The Netherlands; ^3^ Oxford Centre for Functional MRI of the Brain John Radcliffe Hospital Oxford UK

**Keywords:** brain parcellation, causal discovery, Dynamic Causal Modeling, effective connectivity, functional Magnetic Resonance Imaging, neuronal noise

## Abstract

**Purpose:**

Multiple computational studies have demonstrated that essentially all current analytical approaches to determine effective connectivity perform poorly when applied to synthetic functional Magnetic Resonance Imaging (fMRI) datasets. In this study, we take a theoretical approach to investigate the potential factors facilitating and hindering effective connectivity research in fMRI.

**Materials and Methods:**

In this work, we perform a simulation study with use of Dynamic Causal Modeling generative model in order to gain new insights on the influence of factors such as the slow hemodynamic response, mixed signals in the network and short time series, on the effective connectivity estimation in fMRI studies.

**Results:**

First, we perform a Linear Discriminant Analysis study and find that not the hemodynamics itself but mixed signals in the neuronal networks are detrimental to the signatures of distinct connectivity patterns. This result suggests that for statistical methods (which do not involve lagged signals), deconvolving the BOLD responses is not necessary, but at the same time, functional parcellation into Regions of Interest (ROIs) is essential. Second, we study the impact of hemodynamic variability on the inference with use of lagged methods. We find that the local hemodynamic variability provide with an upper bound on the success rate of the lagged methods. Furthermore, we demonstrate that upsampling the data to TRs lower than the TRs in state‐of‐the‐art datasets does not influence the performance of the lagged methods.

**Conclusions:**

Factors such as background scale‐free noise and hemodynamic variability have a major impact on the performance of methods for effective connectivity research in functional Magnetic Resonance Imaging.

## 1 INTRODUCTION

Studies on the communication in large‐scale networks in fMRI were initiated as the *functional* connectivity (FC) research. FC quantifies the strength of communication between brain regions by means of correlation and therefore without specification of directionality (van den Heuvel & Pol, 2010).

Extending network research in fMRI from functional to effective connectivity could provide a substantial advance to the understanding of brain dynamics in health and disease (Bielczyk, Buitelaar, Glennon, & Tiesinga, 2015; Fornito, Zalewsky, & Breakspear, 2015; Friston, 2011; Sporns, 2014). Effective connectivity in fMRI is a complex research problem that involves not only specification of the presence or absence of connections, but also the directionality of the information flow.

There are two main classes of methods for effective connectivity research in fMRI. On one hand, we have structural causal models, which are based on the dependencies between the BOLD time series in different nodes in the networks, without taking time lags into account, for example, Structural Equation Modeling, SEM, (Mclntosh & Gonzalez‐Lima, 1994), Linear Non‐Gaussian Acyclic Models, LiNGAM (Shimizu, Hoyer, Hyvärinen, & Kerminen, 2006) or Bayesian Nets, BNs (Frey & Jojic, 2005). On the other hand, we have state‐space models which infer effective connectivity on the basis of the temporal patterns in the dynamics [e.g., Dynamic Causal Modeling, DCM (Friston, Harrison, & Penny, 2003), Granger Causality, GC, (Granger, 1969; Roebroeck, Formisano, & Goebel, 2011; Seth, Barrett, & Barnett, 2015; Solo, 2016), Transfer Entropy, TE (Lizier, Heinzle, Horstmann, Haynes, & Prokopenko, 2011; Vicente, Wibral, Lindner, & Pipa, 2011)]. There is an ongoing debate upon which class of models is better suited for this research problem (Valdes‐Sosa, Roebroeck, Daunizeau, & Friston, 2011).

Understanding factors influencing performance of methods for effective connectivity research has been a subject to multiple computational studies. Previous work used the Dynamic Causal Modeling generative model (as the basis for the DCM inference procedure (Friston et al., 2003)) to create benchmark synthetic datasets (Smith et al., 2011). Multiple methods for assessing effective connectivity were tested on this synthetic data, including GC (Roebroeck, Formisano, & Goebel, 2005), Partial Directed Coherence (PDC; Baccalá & Sameshima, 2001), LiNGAM (Shimizu et al., 2006) or TE. In general, however, the methods tested in the study did not perform much better than chance even though the testing networks were sparse and relatively small.

In this work, we employ a generative DCM forward model as implemented in (Smith et al., 2011). We then perform a simulation study in order to shed more light on the caveats of effective connectivity studies in fMRI datasets. On the basis of these simulations, we propose how certain problems can be overcome by a proper data preprocessing and region definition.

In Section Materials and Methods: The generative model, we introduce the DCM generative model. In Sections Materials and Methods: Impact of the noise and the length of the signal on identifiability of causal structures in fMRI and Materials and Methods: Influence of hemodynamics on the inference of effective connectivity, we describe in detail how we set up networks in order to perform the Linear Discriminant Analysis (LDA) study and to compute lagged cross correlations, respectively. In the Results section, we present the results and in the Discussion, we discuss these results and their practical implications on the effective connectivity research in fMRI.

## 2 MATERIALS AND METHODS

### 2.1 The generative model

**Figure 1:**
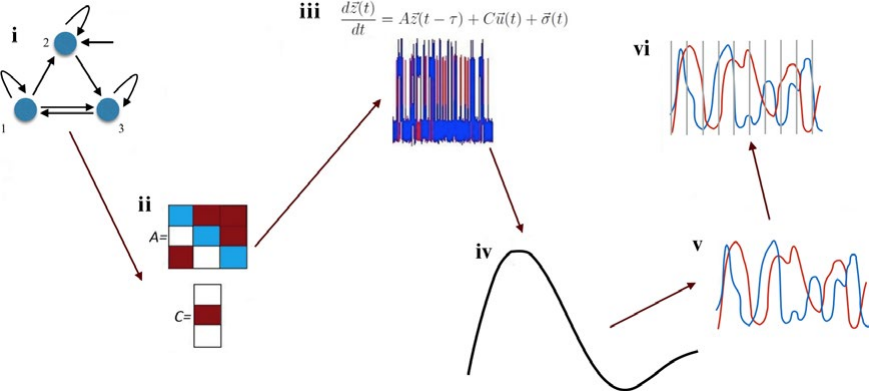
The full pipeline for the Dynamic Causal Modeling forward model. The full parameter set for the network (i) includes adjacency matrix (A) and inputs to the nodes (C) (ii). The neuronal dynamics is generated from this network with use of ordinary differential equations (iii). The neuronal time series is then convolved with the hemodynamic response function (iv) to obtain the BOLD response (v), which may be then (vi) subsampled

Over the past decade, multiple generative models have been proposed in the context of the DCM (Friston, Kahan, Biswal, & Razi, 2011; Friston et al., 2003; Havlicek et al., 2015; Kiebel, Kloppel, Weiskopf, & Friston, 2007; Li et al., 2011; Marreiros, Kiebel, & Friston, 2008; Seth, Chorley, & Barnett, 2013; Smith et al., 2011; Stephan, Weiskopf, Drysdale, Robinson, & Friston, 2007; Stephan et al., 2008). In this study, we chose the original, single‐node per region DCM (Friston et al., 2003; Smith et al., 2011). This model operationalizes the generation of BOLD response from the neuronal networks across two levels: nonobservable neuronal level and the observable hemodynamic level. The latent neuronal dynamics is described by the simple differential relationship:

(1) $$\frac{d\vec{z}\left(t\right)}{dt}=A\vec{z}\left(t-\tau \right)+C\vec{u}\left(t\right)+\vec{\sigma }\left(t\right)$$

where *z*(*t*) denotes the temporary activity across all nodes, *u*(*t*) denotes binary inputs (trains of on‐ and off‐ states in our case), *A* denotes the adjacency matrix of effective connectivity and *C* denotes connections from (experimental) inputs to the nodes, τ denotes a lag in the neuronal communication, and σ(*t*) denotes the level of stochasticity on the neuronal level. [Correction added on 4 August 2017, after first online publication: in Equations 1 and 2, all delta symbol “∂” were changed to letter “*d*”; and both equations have been reformatted to reflect the correct formulas.] In our network setup, the modulatory connectivity does not play a role for the research question, therefore we set all the modulatory connections *B* from the original DCM model (Friston et al., 2003) to zero. The connectivity (a.k.a. adjacency) matrix *A* contains self‐inhibition in every node (Figure 1i) as originally proposed in (Friston et al., 2003). Additionally, we use small, biologically plausible time lags of 50 ms in the communication between areas throughout all simulations in our study (as also implemented in Smith et al., 2011).

Therefore the simulated network becomes a system of delayed differential equations in fact (Bocharov & Rihan, 2000). Furthermore, effective connectivity matrix *A* must fulfill a few additional conditions, listed in the Appendix *Constraints on the adjacency matrix*.

In this context, the stochastic term σ(*t*) represents neuronal innovations which are not the part of the communication between nodes of the investigated network (Daunizeau, Stephan, & Friston, 2012). It can either represent intrinsic dynamics in the given node (other than inhibition), or input from areas outside the investigated network. Strictly speaking, these innovations are not a “noise” (which would mean stochasticity added to the neuronal time series on the top of the simulated dynamics), but rather a background neuronal dynamics which cannot be explained by the given model. However, for the sake of simplicity we will refer to σ(*t*) as noise in the text below.

In this study, we use two versions of the noise: white and scale‐free, pink noise. Pink noise was generated from white noise by applying a Fourier transform, rescaling the spectrum so the power spectral density is proportional to the frequency by factor 1/*f*, and subsequently, applying inverse Fourier transform and normalization.

The observational level is given by the classic model for the hemodynamic response, referred to as Balloon–Windkessel model (Buxton, Wong, & Frank, 1998; Friston et al., 2003), is described node‐wide, and for every node *i* it is described by the dynamics of four biophysiological variables as follows:

(2) $$\begin{array}{cc} & \frac{ds_{i}\left(t\right)}{dt}=z_{i}\left(t\right)-\kappa_{i}s_{i}\left(t\right)-\gamma_{i}\left(f_{i}\left(t\right)-1\right)\\ & \;\;\;\;\frac{df_{i}\left(t\right)}{dt}=s_{i}\left(t\right)\\ & \lambda_{i}\frac{dv_{i}\left(t\right)}{dt}=f_{i}\left(t\right)-{v_{i}}^{\frac{1}{\alpha }}\left(t\right)\\ & \lambda_{i}\frac{dq_{i}\left(t\right)}{dt}=f_{i}\left(t\right)\frac{E(f_{i}\left(t\right),\rho_{i})}{\rho_{i}}-v_{i}^{\frac{1}{\alpha }-1}\left(t\right)q_{i}\left(t\right) \end{array}$$

where *s*_*i*_(*t*)—vasodilatory signal, *f*_*i*_(*t*)—inflow, *v*_*i*_(*t*)—blood volume, *q*_*i*_(*t*)—deoxyhemoglobin content, *E*(*f,* ρ) = 1 *−* (1 *−* ρ)^1/*f*.^ The model involves five node‐specific constants: κ—rate of signal decay, γ—rate of flow‐dependent elimination, λ—hemodynamic transit time, α—Grubb's exponent, ρ—resting oxygen extraction fraction. Then, the following expression describes the outcome BOLD response:

(3) $$y\left(t\right)\;=\;V_{0}\left(7\rho_{i}\left(1\;-\;q_{i}\left(t\right)\right)\;+\;2\left(1\;-\;q_{i}\left(t\right)/v_{i}\left(t\right)\right)\;+\;\left(2\rho_{i}\;-\;0.2\right)\left(1\;-\;v_{i}\left(t\right)\right)\right)$$

where *V*_0_ = 0.02 denotes the resting blood volume fraction. Inputs to the network were simulated as in (Smith et al., 2011): as independent trains of on‐ and off‐states with time resolution of *TR* = 5 ms. The probability of state switches was governed by a Poissonian process of a mean on‐state duration of 2.5 s, and a mean off‐state duration of 7.5 s.

In order to simulate the natural variability in the human hemodynamics, we sampled the parameters independently for each node, and from the distributions described previously in (Friston et al., 2003). Since this work concerns the effects of the neuronal noise, we did *not* add the thermal noise to the hemodynamic response as it refers to the quality of the scanning.

The simulations of the DCM generative model were performed with a step of 5 ms. At the end of the modeling pipeline, we subsampled the BOLD in order to emulate true restrictions of the fMRI datasets. The TR was one of the parameters in our study, with which we also controlled the length of the signal. In the typical fMRI experiments, the range of TR is 0.7 *− *3.0 s, but we made a step beyond this range in order to better understand the influence of the TR on presence of networks signatures in the dynamics, and reduced it down to 0.10 s. The whole pipeline for the data generation with use of the DCM forward model is presented in Figure 1.

### 2.2 Impact of the noise and the length of the signal on identifiability of causal structures in fMRI

In order to investigate under what conditions the problem of effective connectivity research becomes ill‐posed, we fixed a test network and perturbed the connectivity within this network in order to investigate under what circumstances this perturbation yields detectable effects in the outcome BOLD response. Namely, if the two networks of distinct connectivity patterns yield indistinguishable BOLD response, the effective connectivity problem is ill‐posed.

**Figure 2:**
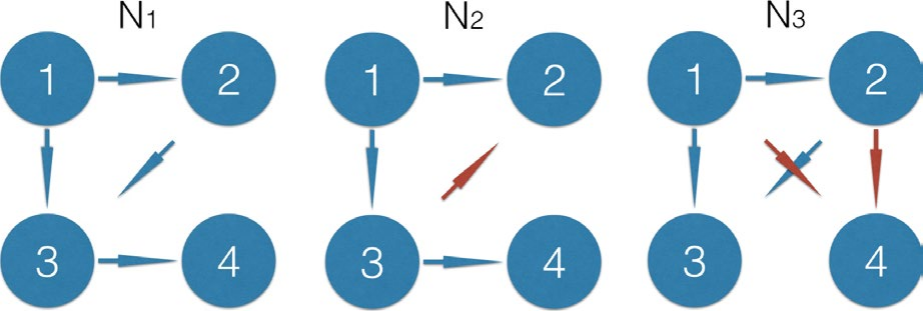
The test network and its two perturbations. The original network (*N*_1_) contains projections 1→2, 1→ 3, 2→ 3, 3→ 4. The connectivity flip involves exchanging the connection 2→3 into 3→2 (*N*_2_, red). The connectivity split involves substituting the connection 3→4 into two weaker connections 1→4 and 2→4 (*N*_3_, red). This is an extension of the two‐node setup presented in Figure 3 to four nodes (inputs and background noise are skipped form the picture for simplicity). The dynamics generated from these networks is presented in Supplementary Material The dynamics for the four‐node DAG with perturbations

In this work, we restricted ourselves to network design comparable to networks investigated in typical fMRI DCM papers. These studies typically involve comparing between small, literature‐informed models of 3*–*4 nodes. We also chose for simple Directed Acyclic Graphs (DAGs; Thulasiraman & Swamy, 1992) presented in Figure 2. This architecture facilitated following the feed‐forward distribution of information throughout the hierarchical connectivity pattern. In Figure 2, the three connectivity patterns proposed in this study are presented. The original network (*N*_1_) contains the projections 1*→*2, 1*→*3, 2*→*3, 3*→*4. We perturbed this original connectivity pattern in two ways:

1. “flip”: exchanging the connection 2*→*3 into 3*→*2 (Figure 2, *N*_2_)
2. “split”: substituting the connection 3*→* 4 into two connections 1*→* 4 and 2*→* 4 (Figure 2, *N*_3_)

Here, we fixed the connectivity strength to 0.15 which refers to connection strengths typically found in the DCM studies (Li et al., 2014; Volza, Eickhoff, Pool, Fink, & Grefkes, 2015).

In this study, we concentrated on the neuronal noise, or “innovations” *z*σ(*t*): other neuronal activity within a node which is either related to intrinsic dynamics of the brain region represented by that node, or to inflow of activity from other regions lying outside this particular network. In a previous study by (Smith et al., 2011), this noise was set to very low levels (namely, to the Gaussian white noise of a standard deviation equal to 0.05 of the high input value), whereas in our study, the magnitude of this noise is the variable of interest. We varied this parameter in the simulations between 0.01 and 2 times the value of the high input state (which corresponds to signal‐to‐noise ratio, SNR = 100 and SNR = 0.50, respectively). Also, as we were interested in quantifying the strength of the effect of mixed signals in the network on the effective connectivity research, we emulated mixed signals with scale‐free, pink noise in the neuronal communication σ(*t*), and compared against the usual Gaussian white noise of the same magnitude. Since the DCM generative model was stochastic in our study, we performed 500 instantiations of the network dynamics for each parameter set.

In order to find out under what circumstances one can properly distinguish the original and perturbed networks, we performed a LDA study (Fisher, 1936). In LDA, a classifier supplied with a labeled training dataset, learns a linear combination of features that best separates the data into given classes. That performance can be then validated on a separate testing dataset. In our study, we first compressed the four‐node time series into six pairwise Pearson correlations. We chose correlations as features because multiple methods for effective connectivity research utilize correlations between the time series. The classification performance was evaluated by cross validation: for each set of 500 instantiations of the networks, the data were randomly assigned to the training‐ (400 networks) and the testing set (100 networks), a 100 times. We then computed the mean performance and the standard deviation over all the random assignments.

Then, we investigated the impact of the magnitude and spectral properties of the noise on the neuronal level and the length of the time series, on the classification accuracy.

### 2.3 Influence of hemodynamics on the inference of effective connectivity

**Figure 3:**
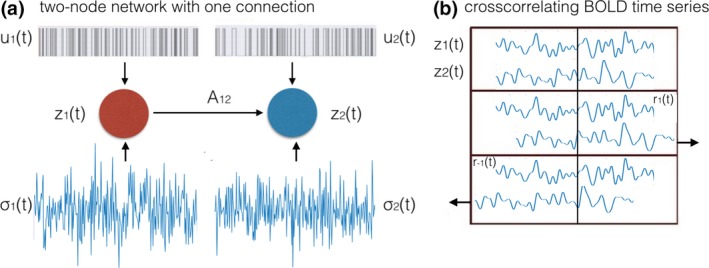
Defining ∆ for the two‐node noisy system. (a) The upstream node [*z*_1_(*t*)] is sending information to the downstream node [*z*_2_(*t*)] through a single connection of weight *A*_12_. Both regions received a binary signal *u*_*i*_(*t*) and neuronal noise σ_*i*_(*t*). (b) Computing cross correlation between the two time series. Correlation between two time series can be computed without a lag (upper panel), with time series generated in the downstream region shifted one sample ahead in time (*r*_1_, middle panel), or backwards in time (*r*_*−*1_, lower panel)

Second, we studied the particular case of lagged methods for effective connectivity. Lagged methods such as GC, TE and other, new approaches (Hyvärinen, Shimizu, & Hoyer, 2008) assume that there is information preserved in the sequence of the BOLD samples. Therefore, we were testing the limitations of the lagged methods for effective connectivity research with respect to the variability in the underlying dynamics and the properties of the local hemodynamic responses. With use of the DCM generative model (Section Materials and Methods: The generative model), we simulated the dynamics of a simple, two‐node system (Figure 3a). Then, we adapted the lagged cross correlation (as proposed in El‐Gohary & McNames, 2007), and proposed a simple quantity ∆ based on asymmetry of the lagged cross correlation (Figure 3b), in order to quantify the amount of information preserved on effective connectivity contained in the *sequence* of the samples. Since in our simple model, node 2 received information from node 1 and the neuronal dynamics was delayed by 50 ms, in the absence of hemodynamic variability the BOLD time series in node 2 was delayed with respect to the BOLD time series in node 1. Let us now define correlation between the activity in node 1, *z*_1_(*t*), and activity in node 2 shifted one sample forward in time, *z*_2_(*t *+* *1), as *r*_1_ (Figure 3b, middle row). Similarly, let us define correlation between the activity in node 1, *z*_1_(*t*), and activity in node 2 shifted one sample backwards in time, *z*_2_(*t − *1), as *r*_−1_ (Figure 3b, bottom row).

Therefore, the BOLD time series in node 2 shifted one sample forward in time *z*_2_(*t *+* *1) (*r*_1_, middle row) correlated with the BOLD time series *z*_1_(*t*) *higher* than the BOLD time series in node 2 shifted one sample backwards in time *z*_2_(*t − *1) (*r*_−1_, bottom row). On the basis of this expected difference, we proposed the variable ∆ = *r*_1_ *− r*_−1_, and we expected this quantity to be positive for the connection 1*→*2. Now, since in the absence of hemodynamic variability between the upstream and the downstream node, we expected *r*_1_ *> r*_−1_, the value of ∆ for the connection 1*→*2 should be positive. We then introduced the variability in the hemodynamic lags and investigated whether the positive sign of ∆ still holds. We also investigated how the TR influences the performance of effective connectivity research with use of ∆, and whether or not further improving the TR to levels lower than 0.70 s as implemented in the state‐of‐the‐art HCP data (van Essen et al., 2013) would improve the performance of the lagged based methods. Therefore, we compared the results for TR = 0.70 s with TR = 0.10 s.

Since in this part of the study, we were only focused on the variability in the hemodynamic lags as a confound to the effective connectivity research, we have set the level of neuronal noise to low magnitudes in both nodes (standard deviation, STD = 0.01, white Gaussian noise), we have set the connectivity strength to a very high value of *w* = 0.9 and we performed a single but long simulation (*T* = 3*,* 600 s) of the neuronal dynamics of this two‐node system. Then, we convolved the neuronal time series in the upstream node [*z*_1_(*t*)] with a *fixed* BOLD response (hemodynamic parameters at the mean of the distributions given in (Friston et al., 2003), which gives a hemodynamic lag of 3.14 s. We used 60,000 different BOLD responses to convolve the downstream neuronal time series *z*_2_(*t*) with. We then derived the ∆ value for all pairs of BOLD time series while assuming TR = 0.70 s (which refers to the state‐of‐the‐art Human Connectome Project datasets), and a very high time resolution of TR = 0.10 s for comparison.

## 3 RESULTS

### 3.1 Impact of the noise, the hemodynamics and the length of the signal on the presence of the network signatures in the BOLD

**Figure 4:**
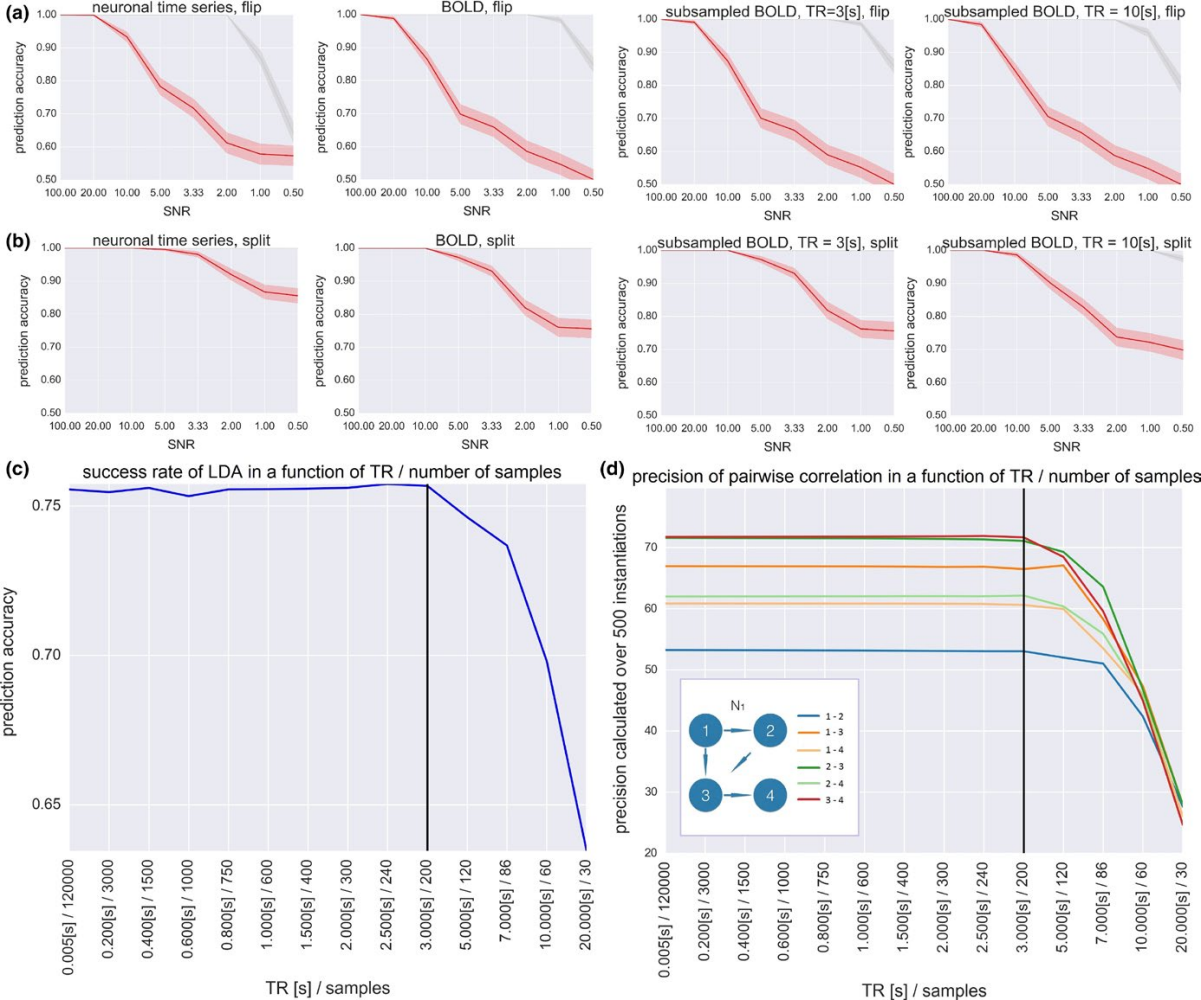
Results of the classification study with the Linear Discriminant Analysis. (a) Neuronal time series, BOLD and BOLD subsampled with two different TRs for the flipped connection. Gray line: white Gaussian noise in the neuronal communication. Red line: pink noise in the neuronal communication. Shaded error bars denote the standard deviation of the Linear Discriminant Analysis (LDA) results over 500 simulations. (b) The same for the split connection. (c) An example demonstrating the dependence of the LDA results on the TR and the number of samples, for fixed combination of parameters: full BOLD time series, pink noise of SNR = 0.5, split connection. TR = 3.0 s is the critical value of TR which gives performance comparable to the full BOLD time series. (d) Precision of the six pairwise correlations in the function of the TR and the number of samples, for fixed combination of parameters: the original network connectivity pattern *N*_1_, the full length BOLD time series, pink noise of SNR = 0.5. The precision in correlation decreases along with decreasing length of the time series

The results of the classification study with LDA are presented in Figure 4a,b. In panel a, we present the results for the flipped connection: the neuronal time series, BOLD and BOLD subsampled with two different TRs (3.0 s and 10.0 s), for the white noise (gray) and the pink noise (red) case. We present the classification accuracy in the *y*‐axis against the SNR in the *x*‐axis. In panel b, we present the results for the split connection, accordingly. In panel c, we present an example demonstrating the dependence of the LDA results on the TR and the number of samples in the time series. In panel d, we present the precision of the six pairwise correlations in the function of the TR and the number of samples.

First, we observe that the split of connection in the network is easier to detect than the flip. This result is intuitive because perturbing the network with a flip involves direct manipulation of one pairwise correlation (pair 2*–*3), whereas perturbing the network with a split involves direct manipulation of three pairwise correlations (pairs 1*–*4, 2*–*3, and 3*–*4).

Second, the dropout in the accuracy of LDA classifier in a function of SNR is similar for the neuronal time series and for the full length BOLD time series. This result suggests that, while using the methods of effective connectivity that are based on correlations between signals, deconvolving BOLD into the neuronal time series might not be necessary to perform the inference. The hemodynamic response can even be beneficial to effective connectivity research as it removes noise‐related higher frequencies while leaving experiment‐related lower frequencies intact.

However, this effect does not hold for the scale‐free pink noise: the hemodynamic convolution can no longer filter out the noise which has high power in the slow frequency range. Importantly, subsampling BOLD time series from very low time step used in the simulations of the generative model (5 ms, which gives a total of 120,000 samples for the 10 min of simulation) into the low TR = 3.0 s resolution (which gives only 200 samples) does not effectively blur the network signatures contained in the pairwise correlations, since LDA performs classification with almost identical accuracy for the BOLD time series subsampled with TR = 3.0 s as for the full‐length BOLD time series obtained from convolving the fast neuronal time series (Figure 4, middle columns). This result can be explained by plotting the success rate of LDA classification for fixed set of parameters: the full BOLD time series, pink noise of SNR = 0.5, split, in the function of the TR (Figure 4c). In this case, TR = 3.0 s is the critical value of TR which gives performance comparable to the full BOLD time series. This TR relates to the total of 200 samples. For the TRs longer than 3.0 s, however, the performance of LDA gradually drops. This can be explained by two factors. First, the precision in (all six) pairwise correlations over 500 instantiations of the network in a function of TR (Figure 4d)—which is in fact the function of the length of the time series. All the six precision functions are constant until the TR reaches a critical value of *TR* = 3.0 s, and above that value, the precision in pairwise correlations drops. Second, TRs longer than 3.0 s give a sampling rate below the Nyquist frequency for the hemodynamic response, which also influences the amount of information contained in the time series. To sum up, given the small network of four nodes with connectivity weights in the range of 0.15, the BOLD time series of about 200 samples should be *long enough* to estimate pairwise correlations between the nodes, and as these are the features fed into the classifier, it is long enough to distinguish between different connectivity patterns.

### 3.2 Influence of hemodynamics on effective connectivity research

**Figure 5:**
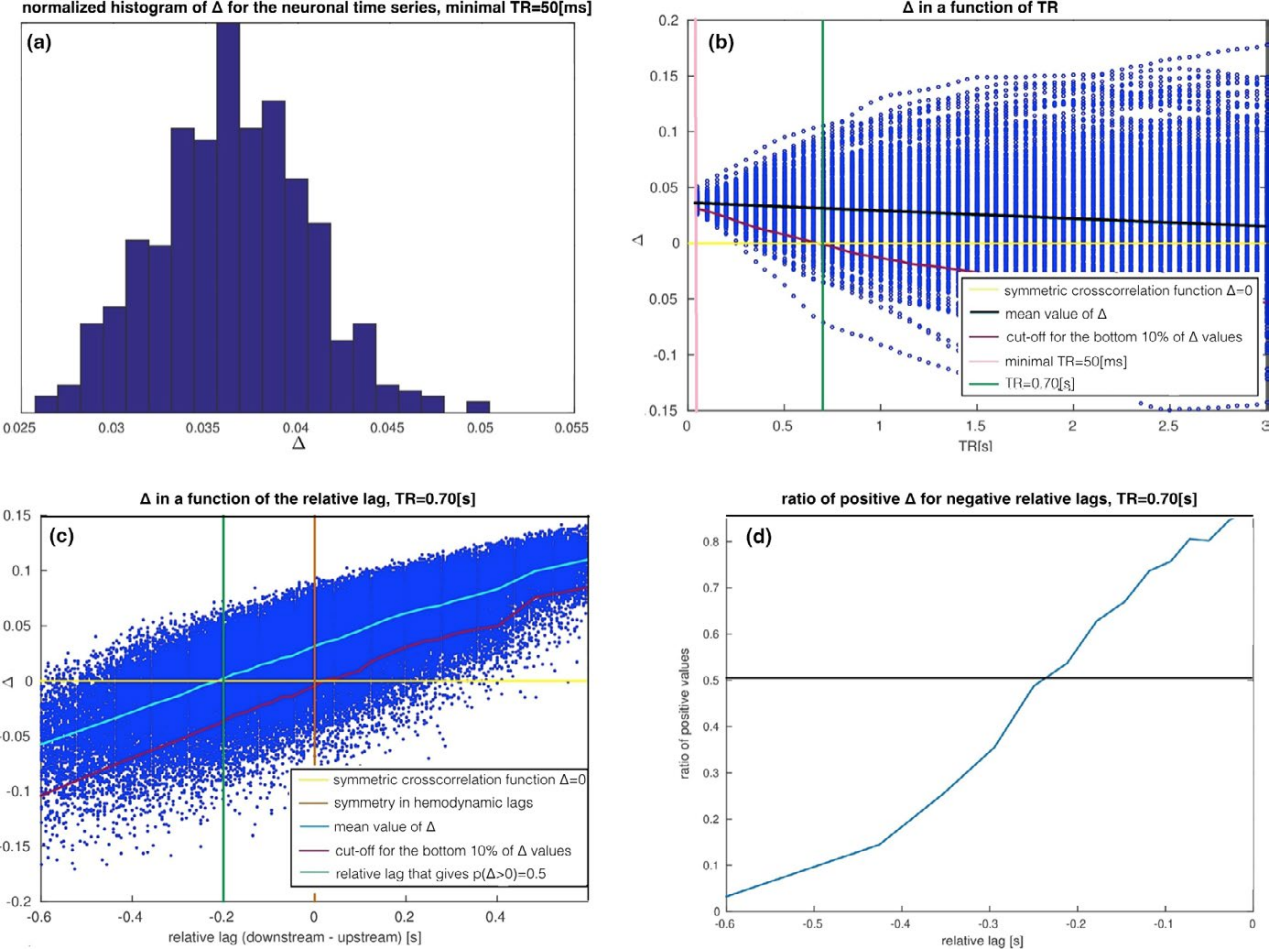
Impact of the relative hemodynamic lags computed as a difference between time to peak of the hemodynamic response in the upstream and downstream region on ∆. (a) The histogram of ∆ values over 500 instantiations of the neuronal dynamics, for the optimal TR = 50 ms matching the true delay in the neuronal communication, gives only positive values as expected. (b) ∆ over 500 instantiations of the neuronal dynamics and in a function of the TR. At TR = 0.70 s, the percentage of positive ∆ values drops down to 90%, which is still high over the chance level. (c) ∆ in the function of the *difference* between hemodynamic lags in the upstream and the downstream region (“relative lag”). (d) The relationship between the percentage of positive ∆ values and the relative lags

In Figure 5, we demonstrate the results of testing ∆ on the two‐node system with varying hemodynamic lags in the downstream region. Hemodynamic lags are operationalized as the time to peak of the hemodynamic response.

In Figure 5a, the histogram of ∆ values over 500 instantiations of the *neuronal dynamics* of a two‐node network with one connection (Figure 3) is presented. Indeed, all values are positive. In Figure 5b, values of ∆ for all TRs between 50 ms and 3 s and over 500 instantiations of the neuronal dynamics are presented. As expected, both the mean value and precision of ∆ drop toward zero along with growing TR (black line). At the point of TR = 0.70 s (green line), 90% of ∆ values are still positive (magenta curve). This means that in the absence of any hemodynamic lags and at the TR = 0.70 s, ∆ would have empirical accuracy rate of 90%.

In Figure 5c, the results for the analysis on the hemodynamic level are presented: ∆ is expressed as the function of the *difference* between hemodynamic lags in the upstream and the downstream region (referred to as a “relative lag”). The plot presents scatter plot over 60,000 different convolutions with hemodynamic parameters independently sampled from the distributions given in (Friston et al., 2003). Since the hemodynamic lag in the downstream node can be either higher or lower than the reference hemodynamic lag in the upstream node, the relative lags have positive and negative values (equal lags are marked with orange line). We divided the whole set of relative lags into 30 intervals, and computed the mean for relative lags lying within each interval (Figure 5c, light blue curve). For each of the intervals, we also computed the cut‐off for the bottom 10% of all the values (magenta curve).

First, for positive relative lags ∆ becomes highly indicative for the connection as the vast majority of the ∆ values are positive. This is because in this case, next to the neuronal dynamics in the downstream region following the dynamics in the upstream region, the slower hemodynamics further increases the lag in the downstream BOLD time series. The upper 90% of the ∆ values (magenta curve) at the relative lag of zero (orange line) is positive (yellow line), which corresponds with the previous results for the underlying neuronal dynamics (Figure 5b).

The negative relative lags on the other hand make this statistic worse: the lower the value of the relative lag, the higher the percentage of ∆ values that become negative, the higher the chance of inferring wrong directionality of the link between the two nodes. Figure 5d demonstrates this relationship. For the critical value of the relative lag of around *−*0.22 s, the percentage of positive ∆ values drops below 50%.

**Figure 6:**
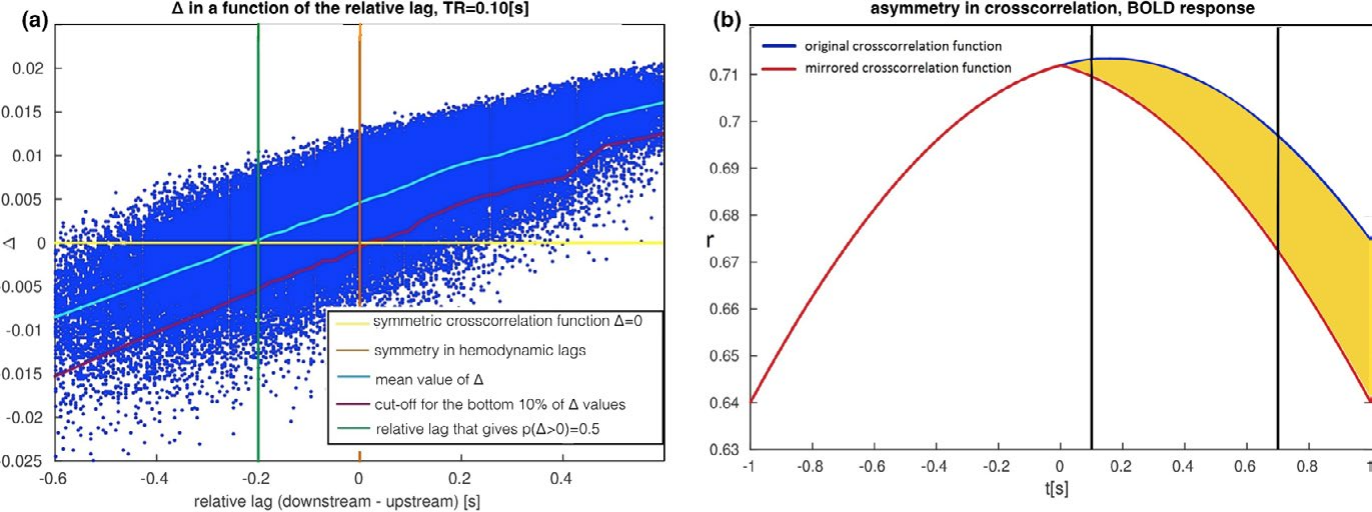
Impact of TR on ∆. (a) A reproduction of Figure 5c but with a small TR = 0.10 s. The absolute values of ∆ change into narrower range than for TR = 0.70 s, but the sign of ∆ stays the same. (b) Dependence of ∆ on low TRs. The difference between cross‐correlation function (blue curve) and its version mirrored around zero (red curve) for *t* = *TR* equals ∆ (yellow area). As we decrease TR from 0.70 s to 0.10 s (black lines), we can observe that TR has impact on the absolute value, but not on the sign of ∆

To conclude, we consider the impact of the TR on these results. In Figure 6a, we demonstrate a reproduction of Figure 5c but with a low TR = 0.10 s. Despite much faster temporal sampling, the results of the lag differences are almost identical, and the main difference being the range of absolute values of ∆ (on the *y* axis). Therefore, the difference is quantitative rather than qualitative. This effectively means that at TR = 0.70 s, the hemodynamics is captured sufficiently well so that lowering TR further does not add any extra information that could be utilized with lagged methods for effective connectivity represented by ∆. This effect is explained in Figure 6b, where an exemplary cross‐correlation function between the BOLD response between the upstream and the downstream region is presented (blue curve). Both time series are convolved with the identical, canonical hemodynamic response. The cross‐correlation peaks at 50 ms and then declines as expected. We mirrored the cross‐correlation function around zero (red curve), and ∆ can be computed as the difference between the original value of cross‐correlation and its mirror counterpart (yellow area). TRs of 0.10 and 0.70 s are marked with vertical black lines. In this low range, the TR has an impact on the *absolute value* of ∆, but not on its sign.

## 4 DISCUSSION

As a summary, our findings suggest that

1. In effective connectivity research with use of methods based on correlations, deconvolution of BOLD time series into neuronal time series might not be necessary. As opposed to the distribution‐based methods, in lagged methods the variability in the local hemodynamic response can inverse the direction of a connection, therefore deconvolution of BOLD time series into the neuronal time series is recommended
2. Scale‐free, pink noise induced by misparcellation is detrimental to the signatures of distinct connectivity patterns in the BOLD time series, therefore functional parcellation into ROIs (e.g., by Bayesian clustering or hierarchical ICA) is recommended
3. Decreasing the TR from 0.7 s (as is implemented in HCP datasets (van Essen et al., 2013), would not improve the performance of the lagged methods

Our LDA prediction study sheds the light on the factors influencing effective connectivity research in fMRI. One remark is that LDA does not indicate *how* to establish the causal relations between the nodes in the network: it just uses the distinct features in the data and therefore, determines when it is theoretically possible. In our study, we chose pairwise correlations as features because Pearson correlation is nonparametric and in multiple methods for effective connectivity research in fMRI, it serves as the basis for the effective connectivity research (Hyvärinen & Smith, 2013; Patel, Bowman, & Rilling, 2006). Having that in mind, we compared the impact of the presence of slow hemodynamic response, short time series and mixed signals on the LDA results.

The results of our LDA study suggest that the *fixed* hemodynamics is not detrimental to the network signatures based on pairwise correlations: the LDA classifier distinguishes networks as easily on the basis on the neuronal time series as on the basis of the BOLD. Moreover, the hemodynamic response can work as a denoiser for the white noise (Figure 4a,b, gray lines). This result is intuitive once we take the spectrum of the white noise into account: a big portion of the power is carried within the high‐frequency range which is easily denoised with the hemodynamic response. This result demonstrates that, in some cases (namely, when the signal has low frequency, and at the same time a good portion of the power of the noise is in the high‐frequency range, and the hemodynamic response is fixed), the BOLD response is not necessarily a detrimental factor to the effective connectivity research. Therefore, according to our results, deconvolving the BOLD time series is not necessary while using methods for effective connectivity based on correlations.

We also obtain an intuitive result linking to the success rate of the LDA classifier to the precision in features estimation. Due to our results, at certain length of the BOLD time series, the precision in estimating pairwise correlations saturates, therefore further increasing the length of the time series does not improve the classification accuracy any further. This is a different characteristic from the relationship between the length of the time series and the accuracy of *signal detection*, where increasing the length of the time series tends to improve the performance, as it was found in a theoretical study by Murphy, Bodurka, & Bandettini (2007).

Furthermore, unlike slow hemodynamics and short time series, mixing signals represented in our simulations by the transition from white to scale‐free (pink) noise, hinders the effective connectivity research in fMRI. For this reason, studying scale‐free noise and its role for the inference with the DCM—as opposed to the Wiener process typically implemented in the stochastic DCM (Daunizeau et al., 2012)—is worth considering. Furthermore, the effect of mixed signals can to some extent be controlled. There are two sources of signal mixing which partly can be addressed by the researcher during data preprocessing and before applying any method for effective connectivity research to the fMRI data. The first source is associated with the background neuronal activity in the networks. In order to control for this possible confound effect, one can *partial out* the signal coming from outside the chosen pair of ROIs as an initial step to the effective connectivity study. The second source of the scale‐free noise is a possible misparcellation into arbitrary brain areas during the preprocessing pipeline. From the perspective of network analysis, mixing signals of various frequencies is equivalent to inducing a pink noise in the underlying neuronal dynamics (Bak, Tang, & Wiesenfeld, 1987). Therefore, one should pursue efforts aiming at functional (as opposed to anatomical) segregation into ROIs. Most of the recent open‐source fMRI datasets such as Human Connectome Project, ADHD‐200 or ABIDE, already support the functional parcellation of the data (Craddock, James, Holtzheimer, Hu, & Mayberg, 2012; Glasser et al., 2016; Rosenberg et al., 2016). Multiple functional parcellations are available in the field of fMRI (Bellec et al., 2006; Bellec, Rosa‐Neto, Lyttelton, Benali, & Evans, 2010; Blumensath et al., 2013; Chen et al., 2013; Craddock et al., 2012; Eickhoff et al., 2011; Flandin et al., 2002; Glasser et al., 2016; Golland, Golland, & Malach, 2007; Janssen, Jylänki, Kessels, & van Gerven, 2015; Janssen, Jylänki, & van Gerven, 2016; Kahnt, Chang, Park, Heinzle, & Haynes, 2012; Lashkari et al., 2012; Lashkari, Vul, Kanwisher, & Golland, 2010; Michel et al., 2012; Orban et al., 2014; Thirion et al., 2006; Tucholka et al., 2008; van den Heuvel, Mandl, & Pol, 2008; Yeo et al., 2011), and the issue of optimal functional parcellation is broadly discussed in the field (Stanley et al., 2013). In particular, in cognitive paradigms, the ROIs can be built in a data‐driven way and on the basis of the patterns of activation only (task localizers, Fedorenko, Hsieh, Nieto‐Castañón, Whitfield‐Gabrieli, & Kanwisher, 2010; Heinzle, Wenzel, & Haynes, 2012).

Second, once we chose ROIs, the effect of mixing signals can be further reduced by a proper signal extraction from ROIs. One possibility is to consider only the first eigenvariate within the anatomical ROIs as proposed by Sato et al. (2010) (and as is implemented in the original version of the DCM inference procedure (Friston et al., 2003) instead of *averaging* activity over the voxels within an ROI.

Finally, our results suggest that once the effect of mixed signals is under control (so that only white noise is present in the neuronal dynamics), the signatures of distinct connectivity patterns are present in the BOLD time series even in a high noise regime. This may encourage further endeavors in the search for new markers of directed connections between brain regions from fMRI data.

In the second part of our study, we utilize lagged cross correlation in order to determine under what conditions the effective connectivity‐related information is preserved in the sequence of the samples and could be retrieved with the lagged methods. The main difference between our implementation of the hemodynamic variability and the previous studies is that we concentrate on the *time to peak* of the hemodynamic response as the representation of the hemodynamic lag, as opposed to the *time to onset* of the hemodynamic response as in (Smith et al., 2011). If the only difference between two hemodynamic responses was the time to onset, then a lag of 50 ms in the underlying neuronal communication would imply that the time to onset in the hemodynamic response in the upstream region 50 ms later than the time to onset in the hemodynamic response in the downstream region would automatically flip the sign of ∆ and therefore also the outcome of the inference. However, this definition of the hemodynamic lags contains an assumption that hemodynamic response is equal to zero for the initial period of time. Such response profiles, however, are not biophysically plausible as in reality, the hemodynamic response has a positive derivative already at the start (Figure 1C, see Section Materials and Methods: The generative model for equations). In our study we employed the classic Balloon–Windkessel model (Buxton et al., 1998; Friston et al., 2003) in order to generate a natural distribution of the hemodynamic responses derived from neurophysiological experiments, and we operationalized hemodynamic lags as the *time to peak* instead. We found that within certain range of the relative hemodynamic lags, the asymmetry in the lagged cross correlation carries information about effective connectivity that can be derived from two BOLD time series.

In our setup, we use a fixed neuronal delay of 50 ms, whereas in another computational study on the influence of hemodynamic response on GC by (Witt & Meyerand, 2009), this variable was a parameter of interest. In our study, neuronal lags higher than 50 ms would increase the asymmetry of the ∆ in a function of relative lags on behalf of the positive relative lags. However, we motivate this small neuronal lag by experimental evidence suggesting that the axonal transmission, as the slowest phase of the neuronal transmission, involves time delays in the range of dozens of milliseconds (Sabatini & Regehr, 1999).

In many applications, lagged methods for effective connectivity in fMRI are applied to the deconvolved BOLD time series, with an example of GC (David et al., 2008; Goodyear et al., 2016; Hutcheson et al., 2015; Ryali, Supekar, Chen, & Menon, 2011; Ryali et al., 2016; Sathian, Deshpande, & Stilla, 2013; Wheelock et al., 2014). However, as demonstrated in Figure 5b, the natural variability in the neuronal dynamics results with an upper bound on the accuracy of the lagged based methods: even assuming a perfect deconvolution (which is never the case in practice), which would allow for perfect retrieval of the neuronal time series from the BOLD time series, for the TR = 0.70 s, the accuracy rate of ∆ would be <100% (for the parameter space we are exploring in this study, this accuracy would be on the level of 90%). This result suggests that it might be worth considering to perform the GC analysis voxel‐wise, and to average the resulting GC over the whole ROI instead of computing the GC ROI‐wise as is often done in the GC studies (Chen et al., 2017; Deshpande, Hu, Stilla, & Sathian, 2008; Goodyear et al., 2016; Regner et al., 2016; Yang et al., 2017). After voxel‐wise application of GC, the result can be averaged over all voxel‐wise GC scores between the two regions. This voxel‐wise application of GC can be performed in multiple ways: the GC score can be averaged with LASSO regularization (Tang, Bressler, Sylvester, Shulman, & Corbetta, 2012), through a multivoxel pattern‐based causality mapping as proposed by Kim, Kim, Ahmad, & Park (2013), or through hierarchical clustering as proposed by Deshpande, LaConte, James, Peltier, & Hu (2009). This strategic twist is already being used in some studies (Katwal, Gore, Gatenby, & Rogers, 2013; Zhao et al., 2016), however, it is not the most common approach in the field yet [voxel‐wise modeling is, however, increasingly popular for finding activation patterns in cognition from fMRI data (Huth, de Heer, Griffiths, Theunissen, & Gallant, 2016)]. Applying GC voxel‐wise has two major advantages. First, computing the final value of GC as a mean value over GC values derived from a large number of voxels should reduce the natural inaccuracy of the lagged methods when applied to the stochastic neuronal dynamics (Figure 5b). Second, it neutralizes the bias coming from possible inaccuracies in the blind deconvolution algorithms.

We also discuss the influence of the local distribution of the hemodynamic lags present in the investigated networks on the performance of the lagged methods for effective connectivity (Figure 5c,d). This part of the analysis refers to the studies in which the lagged methods are applied to the *unconvolved* BOLD, which is often a practice in GC research (Chen et al., 2017; Regner et al., 2016; Zhao et al., 2016). In such case, the utility of lagged methods in fMRI research depends on the variability in the hemodynamic lags. According to our results, under the assumption that the lag in the neuronal communication is in the range of 50 ms, any inference with lagged methods is possible only if the mean hemodynamic lag between all the voxels within the upstream region are no more than 200 ms higher than the mean hemodynamic lag between all the voxels within the downstream region.

In the work by Handwerker, Ollinger, & D'Esposito (2004), it was found that the variability in the hemodynamic lags *between* subjects is higher than the variability *within* subjects. Furthermore, a study by Menon (2012) has demonstrated a large variation in hemodynamics depending on the vessel sizes in the voxels. Even vasculature changes across cortical layers have been shown to cause around 400 ms differences in hemodynamic time‐to‐peak (Silva & Koretsky, 2002).

This experimentally determined local variability in the hemodynamic lags is higher than the “safe range” found in our study (200 ms). Therefore, further neurophysiological studies on the variability in hemodynamic response across the human brain are necessary to advance our ability to characterize causal interactions from BOLD fMRI data, especially with use of the lagged methods such as GC.

Lastly, our results from this part of the study demonstrate that upsampling the BOLD time series to as little as TR = 0.10 s would *not* significantly improve the ability to retrieve the directionality of a connection with use of the lagged methods as the results differ from the results obtained for TR = 0.70 s only quantitatively, but not qualitatively. Our conclusions are concordant with recent theoretical considerations by Solo (2016), but differ from conclusions derived by Seth et al. (2013), Barnett & Seth (2017)) and Lin et al. (2014), who conclude that GC estimation improves with upsampling. The design of our study is also different than the previous studies. The novelty in our study is that we reduced the effective connectivity research with lagged methods to the simplest possible case. First, we quantify the amount of asymmetry in the lagged cross‐correlation function between two time series, which is a model‐free and probably the simplest lagged method for effective connectivity research (and is precise at picking on the time lag of the interaction, as demonstrated in Supplementary Material *Comparison between lagged cross correlation and Granger Causality*, Figure S1A). Second, we reduce the setup to the simplest, two‐node neural mass model with a single connection, and with neuronal dynamics modeled with ordinary differential equations, as in the standard DCM generative model (Seth et al., 2013), by comparison, use much more complex design, involving a few nodes with more complex, spiking neuronal dynamics). Therefore, we believe that our results give insights into how, in general, a lagged method for rendering effective connectivity can be affected by the neuronal and hemodynamic variability, as well as by the TR.

One last remark is that in this study, we performed a simulation in order to approach the research questions posed in this article, as done in e.g. in Smith et al. (2011). One reason for this choice is that still, little is known about the effective connectivity structures in the human brain, as they are by definition dynamic and context dependent. Multiple ongoing projects aim at establishing effective connectivity in the brain on the basis of TMS and EEG (Esser, 2008) procedures, therefore extending our investigations to experimental fMRI data might be feasible in the future.

Altogether, our results suggest that neither the slow hemodynamics, a relatively short time series of a few hundred samples, nor TR as high as 0.7 s can affect the effective connectivity research in fMRI to a large extent. On the other hand, a proper region definition can facilitate the inference, therefore a good choice of the preprocessing pipeline improves chances for success in the effective connectivity study in fMRI.

## CONFLICT OF INTEREST

The authors declare that the research was conducted in the absence of any commercial or financial relationships that could be construed as a potential conflict of interest. JG has acted as a consultant to Boehringer Ingelheim in the last 3 years, but is not an employee or shareholder of this company.

## AUTHOR CONTRIBUTIONS

NB, AL, and CB designed the study. NB conducted the simulations of the forward model. NB drafted the work. AL, CB, JB, and JG critically revised the work.
