# Supplementary figures and images for: The impact of hemodynamic variability and signal mixing on the identifiability of effective connectivity structures in BOLD fMRI
Source: Brain Behav. 2017 Jul 20;7(8):e00777. doi: 10.1002/brb3.777 (PMC5561328; doi:10.1002/brb3.777)

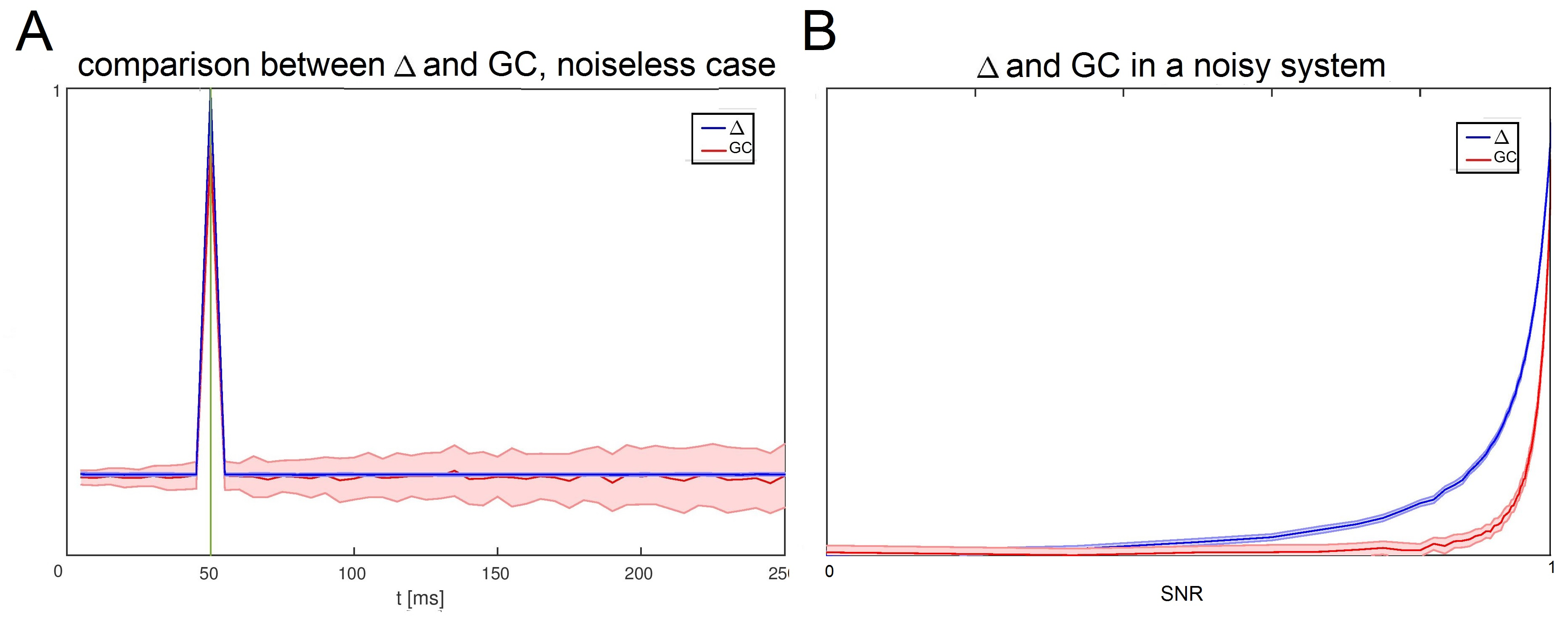

Supplement: Supplementary file 1 [file BRB3-7-e00777-s001.png]

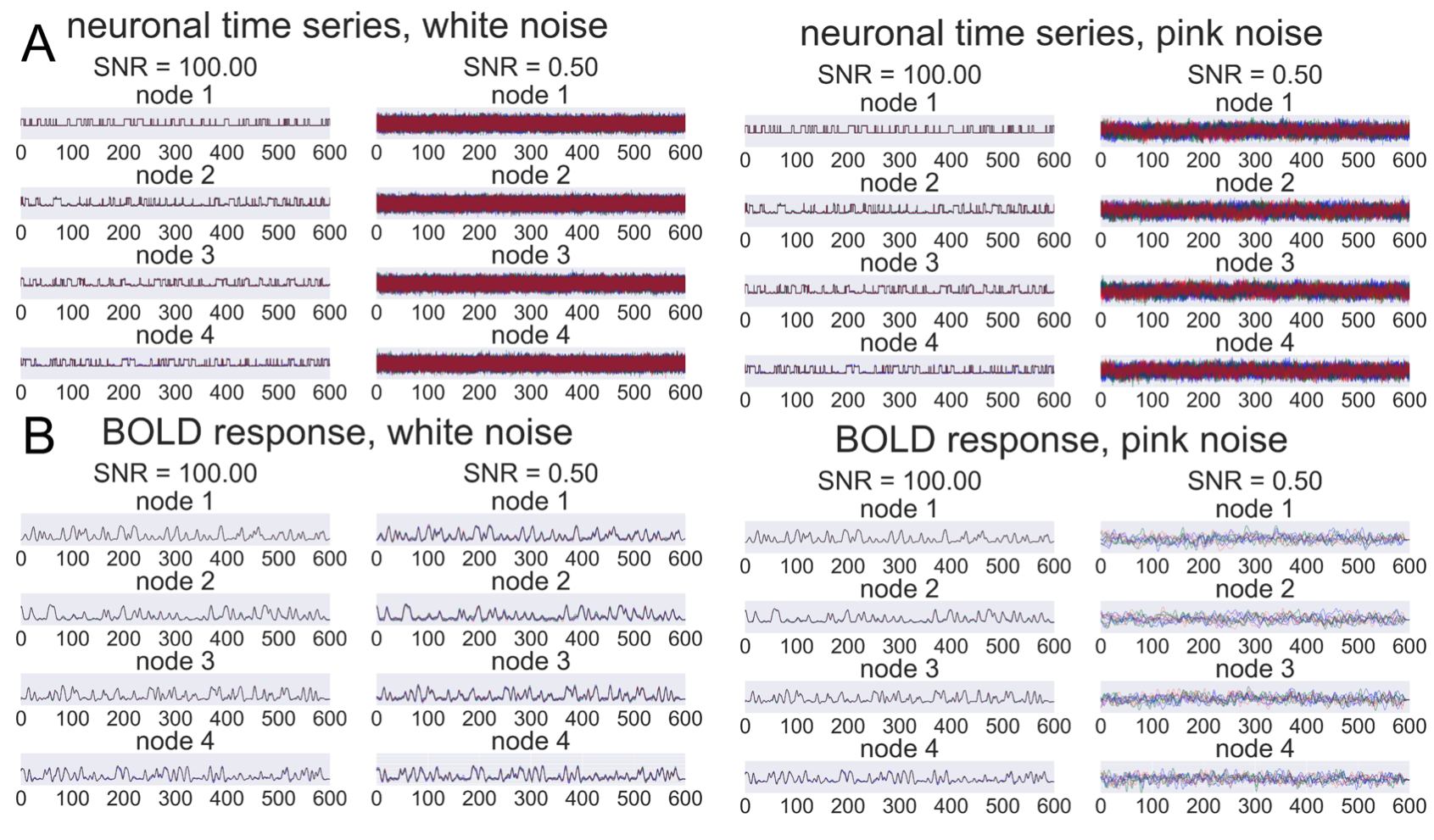

Supplement: Supplementary file 2 [file BRB3-7-e00777-s002.png]

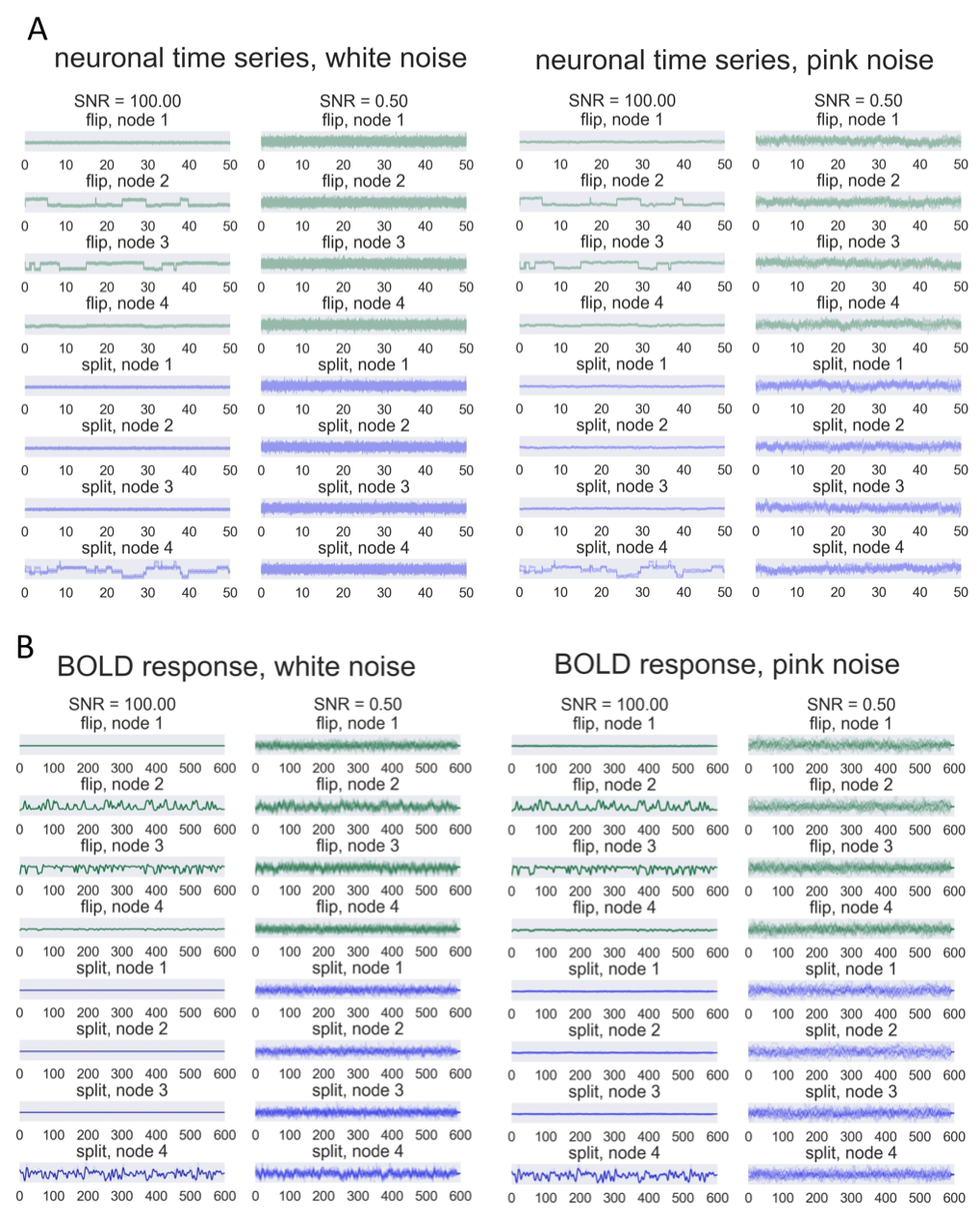

Supplement: Supplementary file 3 [file BRB3-7-e00777-s003.png]
