# Supplementary material for: The impact of hemodynamic variability and signal mixing on the identifiability of effective connectivity structures in BOLD fMRI
Source: Brain Behav. 2017 Jul 20;7(8):e00777. doi: 10.1002/brb3.777 (PMC5561328; doi:10.1002/brb3.777)
Supplement: Supplementary file 4 [file BRB3-7-e00777-s004.docx]

**Supplementary Materials**

**Constraints on the adjacency matrix**

In general, every adjacency matrix *A* which satisfies the following conditions is eligible for our purpose:

a) *A* should guarantee the stable dynamics in the network. Since we imposed the inputs to be binary and low-frequency, we can simplify consider the ODE system describing the neuronal interactions to *x*˙ = *Ax*. The same as in classic DCM model, we assume that self-inhibition in all regions is the same and equals *−*1. Then, for the sake of stability, all we need is a negative real part of all *A*’s eigenvalues, but we also make sure that the ODE is distant enough from the critical dynamics so that the additional stochasticity *σ*(*t*) does not destabilize the system. This also means that, for a given network size and fixed connection weights, only some combinations of a value of connection weight and connection density are ’allowed’ (Figure 2 A, combinations always possible marked in green, combinations possible only for some connectivity patterns marked in yellow)

b) *A* should not contain isolated nodes *j*, such that *∀i/*=*j A*(*i, j*) = 0

c) *A* should contain at least one unidirectional connection in the network *A*(*i, j*) such that *A*(*i, j*) ~= 0 and *A*(*j, i*) = 0 (’flippable’)

**Comparison between lagged crosscorrelation and Granger Causality**

∆ derived from the asymmetry in crosscorrelation functions is meant to be a model-free and computationally efficient method for rendering effective connectivity that can represent the lagged methods in our simulation study. In order to support this choice, in Figure S1 A, we present a comparison between ∆ and GC for 100 instantiations of a two random time series shifted by *τ* = 50[*ms*]. GC was computed with the MVGC software (Barnett & Seth, 2014; Seth et al., 2013). As GC is computed in different units than ∆, the range of values were aligned to each other on the plot. As demonstrated in the figure, ∆ characterizes similar type of information as GC, as the two methods are similarly accurate in picking on the precise lag of the interaction (vertical line). In Figure S1 B, we present the mean value of ∆ and GC at *t* = 50[*ms*] in a function of SNR, for 100 instantiations at each SNR level. The mean value of ∆ decreases slower than the mean value of GC with respect to the decreasing SNR.

# The dynamics for the 4-node DAG with perturbations

Figure S2 presents the dynamics in the 4-node network with perturbations. The signatures of distinct connectivity patterns vanish with growing SNR for the pink but not for the white noise.

Figure S3 presents the residuals between time series generated from original network and network with a flip (green time series) and split (blue time series). For the neuronal time series, we present two realizations (Figure S3 A), and for the BOLD time series, we extend to twenty realizations (Figure S3 B). We present the results for two signal-to-noise ratio levels (*SN R* = 100*.*0 and *SN R* = 0*.*5, which reflects neuronal noise of standard deviation equal to 0*.*01 and 2*.*0 of the magnitude of the high input, respectively), for the white and the pink noise. While SNR is high, for both white and pink noise, one may observe systematic differences between the distributions of neuronal time series and BOLD coming from the original connectivity pattern and its perturbed versions. Once SNR decreases, the results differ with respect to the type of the noise. For the same magnitude of the noise, the dynamics for the pink noise is different than for the white noise. In the neuronal time series, the prevailing component of the dynamics are the slow fluctuations carried by the scale-free, pink noise. The systematic differences between the dynamics in the BOLD generated from the original and perturbed networks are also lost.

**Figure legends:**

*Figure S1* A: the relationship between ∆ = *r*1 *− r−*1, and GC obtained from the standard MVGC software (Barnett & Seth, 2014; Seth et al., 2013), for a hundred instantiations of two random time series shifted by *τ* = 50[*ms*]. As GC is computed in different units than ∆, the range of values were aligned to each other on the plot. ∆ and GC are similarly accurate in picking on the precise lag of the interaction (vertical line). B: The value of ∆ and GC at *t* = 50[*ms*], in a function of SNR. The mean value of ∆ decreases slower than the mean value of GC with respect to the decreasing SNR.

*Figure S2* Exemplary dynamics for a DAG of 4 nodes, for the white and pink noise and across two noise levels. A: neuronal time series. B: BOLD response. Red: original network. Green: network with a flipped connection. Blue: network with a split connection. In nodes 2 and 3, there are certain differences between the dynamics in the original network (red) and network with a flip (green), which reflects the flip of connection from 2 *→* 3 to 3 *→* 2. In node 4, there is a difference between the dynamics in the original network (red) and network with a split (blue), which reflects the split of connection from 3 *→* 4 into 1 *→* 4 and 2 *→* 4. However, the same magnitude of pink noise yields loss of the systematic differences between the dynamics in the considered networks.

*Figure S3* Residuals between signals generated from the original network and from perturbed networks, for the white and pink noise and across two noise levels. A: neuronal time series, 2 realizations. B: BOLD response, 20 realizations. Green: residuum for a flipped connection. Blue: residuum for a split connection. Since neuronal time series has a very fast dynamics, the structure in the residuum (in white noise regime) are not visible even when time interval is shrunk to 50[*s*]. For the BOLD, structure in the residuals are visible both for the flip (nodes 2 and 3) and for the split (node 4) even under low SNR in the white noise regime, but no longer in the pink noise regime.
